# Molecular Mechanism of HER2 Rapid Internalization and Redirected Trafficking Induced by Anti-HER2 Biparatopic Antibody

**DOI:** 10.3390/antib9030049

**Published:** 2020-09-18

**Authors:** Jackie Cheng, Meina Liang, Miguel F. Carvalho, Natalie Tigue, Raffaella Faggioni, Lorin K. Roskos, Inna Vainshtein

**Affiliations:** 1 Integrated Bioanalysis, Clinical Pharmacology and Safety Sciences, R&D, AstraZeneca, South San Francisco, CA 94080, USA; jackie.cheng@precisionformedicine.com (J.C.); meina.liang@astrazeneca.com (M.L.); rfaggioni@exelixis.com (R.F.); lroskos@exelixis.com (L.K.R.); 2 Antibody Discovery & Protein Engineering, BioPharmaceuticals R&D, AstraZeneca, Granta Park, Cambridge CB21 6GH, UK; mfcarvalho@iplantprotect.pt (M.F.C.); natalie.tigue@astrazeneca.com (N.T.)

**Keywords:** HER2, antibody internalization, bispecific, biparatopic antibody, intracellular trafficking, protein degradation

## Abstract

Amplification and overexpression of HER2 (human epidermal growth factor receptor 2), an ErbB2 receptor tyrosine kinase, have been implicated in human cancer and metastasis. A bispecific tetravalent anti-HER2 antibody (anti-HER2-Bs), targeting two non-overlapping epitopes on HER2 in domain IV (trastuzumab) and domain II (39S), has been reported to induce rapid internalization and efficient degradation of HER2 receptors. In this study, we investigated the molecular mechanism of this antibody-induced rapid HER2 internalization and intracellular trafficking. Using quantitative fluorescent imaging, we compared the internalization kinetics of anti-HER2-Bs and its parental arm antibodies, alone or in combinations and under various internalization-promoting conditions. The results demonstrated that concurrent engagement of both epitopes was necessary for rapid anti-HER2-Bs internalization. Cellular uptake of anti-HER2-Bs and parental arm antibodies occurred via clathrin-dependent endocytosis; however, inside the cells antibodies directed different trafficking pathways. Trastuzumab dissociated from HER2 in 2 h, enabling the receptor to recycle, whereas anti-HER2-Bs stayed associated with the receptor throughout the entire endocytic pathway, promoting receptor ubiquitination, trafficking to the lysosomes, and efficient degradation. Consistent with routing HER2 to degradation, anti-HER2-Bs significantly reduced HER2 shedding and altered its exosomal export. Collectively, these results enable a better understanding of the mechanism of action of anti-Her2-Bs and can guide the rational design of anti-HER2 therapeutics as well as other bispecific molecules.

## 1. Introduction

Human epidermal growth factor receptor 2 (HER2/ErbB2/Neu) is a member of the ErbB receptor tyrosine kinase family, which also includes HER1 (epidermal growth factor receptor 1/EGFR/ErbB1), HER3 (ErbB3), and HER4 (ErbB4). Amplification of HER2 has been shown to play a critical role in human cancer malignancies and metastasis [1,2,3]. More than a quarter of metastatic breast cancers have showed an overexpression of HER2 receptors [4,5,6,7,8]. Because of this, HER2 has become an important target for breast cancer therapeutics [9]. HER2-directed therapies, such as anti-HER2 monoclonal antibodies and kinase inhibitors (lapatinib [10]), have been extensively used for the treatment of HER2-positive breast cancer patients [11]. Trastuzumab, a humanized anti-HER2 monoclonal antibody, in combination with chemotherapy is now the standard care treatment for HER2-positive breast cancer patients [12]. Another humanized monoclonal anti-HER2 antibody, pertuzumab, in combination with trastuzumab and docetaxel has been approved for treatment of HER2-positive metastatic breast cancers [13]. Trastuzumab and pertuzumab are monoclonal immunoglobulin G1 (IgG1) antibodies, and they target different domains on the HER2 receptor. Trastuzumab binds to domain IV and blocks ligand-independent HER2-HER2 homodimerization [14], whereas pertuzumab binds to an epitope in domain II and blocks ligand-induced heterodimerization of HER2-HER3 [15]. The combination of trastuzumab and pertuzumab provides an efficient blockade of mitogen-activated protein kinase (MAPK) and phosphoinositide 3-kinase (PI3K) downstream signaling [14,15]. In addition, they also exert antibody-dependent cellular cytotoxicity (ADCC) and complement-dependent cytotoxicity (CDC) as part of their mechanisms of action [16,17,18]. Although anti-HER2 monoclonal antibodies provided efficacy and enhanced progression-free survival for many cancer patients, they have not been efficacious for cancer patients with low HER2 expression in tumors. More importantly, many of the responding patients eventually developed drug resistance leading to disease relapse [19]. To overcome the acquired drug resistance and to address the unmet medical needs, a new class of therapeutics, antibody drug conjugates (ADCs), has recently emerged [20,21]. An ADC is comprised of a target-specific monoclonal antibody linked via a synthetic linker to a potent cytotoxic agent, enabling targeted delivery of the cytotoxic agent to tumor cells. Trastuzumab conjugated to tubulin toxin, mertansine (T-DM1 [22]), is the ADC version of trastuzumab. T-DM1 was originally approved for treatment of HER2-positive metastatic breast cancer [23] with a recent expanded approval for treatment of early stage HER2-positive cancers [24,25,26]. Trastuzumab deruxtecan (T-DXd, formerly DS-8201a), an antibody drug conjugate of trastuzumab with a novel topoisomerase I inhibitor payload, has been recently approved in the US for the treatment of adult patients with unresectable or metastatic HER2-positive breast cancer who have received two or more prior anti-HER2-based regimens in the metastatic setting [27].

To effectively deliver the cytotoxic payload to the target cells, an ADC molecule needs to be internalized into the target cells. Upon delivery, the payloads are released inside the target cell and induce cytotoxic activities. After cell disintegration, the payload can diffuse to neighboring tumor cells, exerting a bystander effect. ADCs that are designed with cleavable or pH-sensitive linkers release their payloads at low pH when the internalized ADC reaches the acidic endosomal compartments. Some ADCs are designed with non-cleavable linkers to minimize possible off-target toxicity [22]. Such molecules need to traffic through the endosomal pathway to reach lysosomes, where the ADCs are degraded and payloads are released. Since the efficacy of an ADC depends on the internalization efficiency and subsequent intracellular trafficking, this information is very important in drug development to estimate the efficacious potential of future ADCs [28,29,30,31,32].

Different HER2 antibodies have been shown to internalize the same receptor with different efficiencies. For example, trastuzumab induced HER2 internalization, albeit not very efficiently [33,34]. By contrast, it was reported that treatment with a polyclonal anti-HER2 antibody induced rapid HER2 internalization [34]. The accelerated HER2 internalization in the presence of the polyclonal anti-HER2 antibody may be due to multi-epitope interactions of the antibodies with the receptor, thus crosslinking HER2 on the cell surface, leading to subsequent efficient endocytosis. Several studies have showed that a combination of antibodies with non-overlapping epitopes can lead to accelerated internalization [35]. For example, the combination of anti-HER2 antibodies trastuzumab (domain IV) and L26 (domain II) resulted in accelerated internalization of the HER2 receptor, tested in SK-BR-3 cells and transfected HEK-293T cells, respectively [36]. In another study, a different combination of anti-HER2 antibodies, N12 and L431, which also contained non-overlapping epitopes, resulted in accelerated internalization of the HER2 receptor in N87 cells [37]. In addition to inducing rapid internalization, these antibody combinations also altered the intracellular trafficking of the internalized receptors. For example, treatment with anti-HER2 antibodies in combination [36,37] or the polyclonal anti-HER2 antibody [34] induced receptor degradation instead of maintaining HER2 on the cell surface.

The bispecific antibody format has become an attractive design for cancer therapeutics [38,39]. By engineering a bispecific antibody with two distinct epitopes (against either the same or two different targets) on a single molecule, the time and costs for testing in early drug development as well as manufacturing and drug administration may be significantly reduced compared to using antibodies in combination. In addition, the bispecific antibody could be an effective approach for combining drugs to induce synergistic anti-tumor activities and a novel mechanism of action [40,41]. ADC drugs would also benefit from the bispecific design where binding domains of two antibodies are placed in one molecule for efficient payload delivery. To this end, John Li. et al. [42] developed anti-HER2-Bs, a fully human tetravalent biparatopic IgG1 antibody, which targets non-overlapping epitopes in domain IV (trastuzumab) and a novel epitope in domain II (39S) of the HER2 receptor. The molecule was then engineered into an ADC molecule, MEDI4276, which contained four molecules of tubulysin attached to specific sites in the fragment crystallizable Fc region [43]. Treatment with MEDI4276 not only effectively blocked HER2 dimerization and its downstream signaling but also induced efficient cell killing [42]. Although anti-HER2-Bs targeted non-overlapping epitopes in domains II and IV, in contrast to combinations of trastuzumab + L26 or N12 + L431, the combination of the monospecific 39S and trastuzumab antibodies induced neither accelerated HER2 internalization nor degradation. However, when engineered in a bispecific format these anti-HER2 antibodies (anti-HER2-Bs) induced rapid internalization and efficient degradation of the receptor [42]. In this study, we examined the molecular mechanism and critical determinants of anti-HER2-Bs that promoted accelerated HER2 internalization and its rerouted intracellular trafficking. Our findings provide important information for understanding the mechanism of action of anti-HER2-Bs and MEDI4276 and will help the rational design of ADCs to obtain efficacious therapeutics.

## 2. Materials and Methods

### 2.1. Cell Culture

Human mammary carcinoma cell lines BT-474, SK-BR-3, and MCF-7 cells were purchased from American Type Culture Collection (ATCC). BT-474 cells were maintained in RPMI-1640 media (Cat# SH30027.01, Hyclone, GE Healthcare Life Sciences, Chicago, IL, USA) supplemented with 10% fetal bovine serum (FBS, Cat# SH30071.03HI, Hyclone, GE Healthcare Life Sciences). SK-BR-3 cells were maintained in McCoy’s 5A media (Cat# 16600-082, Life Technologies, Carlsbad, CA, USA) supplemented with 10% FBS. MCF-7 were grown and maintained in minimum essential medium (MEM) (ThermoFisher Scientific, Cat# 42360032, Waltham, MA, USA) supplemented with 10% FBS, non-essential amino acids (NEAA) (ThermoFisher Scientific, Cat# 11140050) at 1X, and recombinant human insulin (ThermoFisher Scientific, Cat# 12585014, Waltham, MA, USA) at 10 µg/mL.

### 2.2. Antibodies and Reagents

Anti-human HER2 monoclonal antibodies (trastuzumab and 39S), anti-human HER2 bispecific antibodies (anti-HER2-Bs), and control antibody R347 were made in-house (AstraZeneca, Gaithersburg, MD, USA). Rabbit anti-HER2 (Clone 29D8, Cat# 2165S) and rabbit anti- Glyceraldehyde-3-Phosphate Dehydrogenase (GAPDH) (Clone D16H11, Cat# 5174S) monoclonal antibodies were purchased from Cell Signaling Technology (Danvers, MA, USA). Rabbit anti-human HER2 polyclonal antibody (Cat# A048529-2) was purchased from Dako, Agilent Technologies (Santa Clara, CA, USA). Alexa Fluor 488 (AF488) anti-human LAMP1 antibody (Clone H4A3, Cat# 328610) was purchased from Biolegend (San Diego, CA, USA). AF488 goat anti-rabbit IgG (H+L, Cat# A11034), Carboxyfluorescein Succinimidyl Ester (CFSE, Cat# C34554), high-content screening (HCS) CellMask Blue Stain (Cat# H32720), and StemPro Accutase (Cat# A1110501) were purchased from Life Technologies. Human Fc receptor (FcR) blocking reagent (Cat# 130-059-901) was purchased from Miltenyi Biotec (Sunnyvale, CA, USA). Goat anti-human IgG (H+L, Cat# 109-001-003) and horseradish peroxidase-conjugated donkey anti-rabbit IgG (H+L, Cat# 711-035-152) were purchased from Jackson Immuno Research (West Grove, PA, USA). Dyngo 4a (Cat# ab120689) and mouse anti-CD63 monoclonal antibody (Clone TS63, Cat# ab59479) were purchased from Abcam. Pitstop 2 (Cat# C7487) was purchased from Cellagen Technology (San Diego, CA, USA).

### 2.3. Labeling Monoclonal Antibody with Alexa Fluor Dye

Anti-HER2 antibodies (trastuzumab, 39S and anti-HER2-Bs) were labeled with Alexa Fluor 647 (AF647) using Monoclonal Antibody Labeling Kit (Cat# A20186, Life Technologies) according to the manufacturer’s protocol. Before labeling, the buffer of the antibodies was replaced with phosphate buffered saline PBS using Zeba Spin Desalting Columns, 40K MWCO (Cat# 87768, Thermo Scientific, Waltham, MA, USA). The concentration of the antibodies was adjusted to 1 mg/mL with PBS. One hundred micrograms of antibody was used in each labeling reaction. After labeling, antibody concentration and the degree of labeling were determined by measuring A280 and A650 following the manufacturer’s protocol. The labeling ratio of mole of Alexa Fluor dye per mole of antibody was between 5.6 and 8.4. The Alexa Fluor-conjugated antibodies were stored at 4 °C for 1–2 months.

### 2.4. Cell Staining for Antibody Internalization

A total of 20,000 BT-474, SK-BR-3, or MCF7 cells were seeded into each well in the Opera 384-well CellCarrier plate (Cat# 6007550, PerkinElmer, Waltham, MA, USA) overnight in an incubator. On the next day, the cells were briefly washed with prewarmed serum-free media and then incubated with cytoplasm dyes 5 μM CFSE or 5 μM HCS CellMask Blue Stain diluted in the serum-free media for 30 min in the incubator. Unincorporated CFSE dye was removed by a wash with staining media (phenol red-free media supplemented with 1% FBS). Cells were then chilled on ice, blocked with the human FcR blocker reagent (Figure S1) for 15 min, and then stained with AF647-conjugated antibodies for 1 h on ice. After removal of unbound fluorescent antibodies by gentle pipetting, cells were incubated with cold staining media and transferred immediately to PerkinElmer Opera for image acquisition.

For staining in suspension, cells were treated with prewarmed Accutase solution and resuspended with prewarmed serum-free media at a concentration of 3 × 10^6^ cells per mL. The cell suspensions were stained with a similar approach to that for the adherent cells. The cells were washed with serum-free media using centrifugation at 1200 rpm for 7 min at 4 °C. The stained cells were transferred to the Opera 384-well CellCarrier plate and briefly centrifuged at 1000 rpm for 20 s at 4 °C before image acquisition.

### 2.5. Immunofluorescence Staining of Lysosomes and Total HER2 Receptors

To stain for the lysosomes and the total HER2 receptors, cells with internalized antibodies were fixed at the indicated time point with 4% paraformaldehyde (PFA) (diluted from 16% stock with PBS, Cat# RT15710, Electron Microscopy Sciences, Hatfield, PA, USA) in the dark for at least 20 min at room temperature, or overnight at 4 °C. The cells were washed with PBS to remove PFA and then incubated with permeabilization/blocking/staining buffer (PBS supplemented with 0.1% saponin and 2% bovine serum albumin (BSA)) for 30 min at room temperature. Next, the cells were incubated with AF488 anti-LAMP1 antibody or anti-human HER2 polyclonal antibody (Dako), diluted at 1:1000 in permeabilization/blocking/staining buffer, in the dark for 1 h. The cells were then washed twice with PBS. For staining HER2 receptors, the cells were then incubated with AF488 goat anti-rabbit IgG, diluted at 1:1000 in permeabilization/blocking/staining buffer, in the dark for 1 h. After two washes of PBS, the cells were used for image acquisition.

### 2.6. Confocal Imaging with PerkinElmer Opera High-Content System

Before each image acquisition and new experiment, the camera system was aligned with the Opera Adjustment Plate (PerkinElmer, Waltham, MA, USA) to set up skewcrop and reference image analyses properly. To perform live cell kinetic imaging, the live chamber of the Opera system was prewarmed to 37 °C with 5% CO_2_ and 70% humidity. The CellCarrier plate with cells was transferred from an ice pan to the imaging chamber to immediately initiate image acquisition. A series of images were recorded at indicated time intervals. A 60X water immersion objective with a numerical aperture of 1.2 was used.

A combination of excitation lasers, emission filters, and sequential exposure was determined based on fluorophores to maximize signals with minimum possible crosstalk between channels. Prior to live cell imaging acquisition, optimal exposure parameters including laser voltages, exposure times, and image focus height were set up using a separate test plate with the cells stained by the same method. The optimal exposure parameters, the plate layout, and the sublayout were then used in the automatic acquisition mode in the Opera 2.0 software (PerkinElmer). Images were saved in a dedicated image server and subsequently processed using Opera 2.0 software, ImageJ (NIH, Bethesda, MD, USA), and Adobe Illustrator (Adobe, San Jose, CA, USA). For immunofluorescence of fixed cell samples in the CellCarrier plate, images were acquired using a similar approach at room temperature.

### 2.7. Pitstop 2 and Dyngo 4a Treatment

Pitstop 2 and Dyngo 4a were dissolved with DMSO (Cat# D2650, Sigma Aldrich, St. Louis, MO, USA) as a stock solution at 50 mM and stored in aliquots at −20 °C. BT-474 was seeded into the CellCarrier plate overnight as previously described in Section 2.4. On the next day, the cells were pretreated with either 30 μM Pitstop 2 or Dyngo 4a, diluted in RPMI-1640 full media, in the incubator for 2 h. The cells were then stained with cytoplasmic dye and antibody in a similar approach but in the presence of the inhibitor. Pitstop 2 or Dyngo 4a was also added to the staining media during image acquisition.

### 2.8. Immunoprecipitation and Western Blotting

SK-BR-3 cells were seeded into poly-D-lysine-coated 6-well plates and allowed to grow in an incubator overnight. On the next day, the cells were washed with prewarmed serum-free MEM media before incubating with 10 μM MG-132 (474791, Millipore, Hayward, CA, USA) diluted in prewarmed serum-free MEM for 2 h in the incubator. The cells were then treated with 10 μg/mL anti-HER2-Bs in the presence of MG-132 for different time periods in the incubator. After the treatment, the cells were washed once with ice-cold PBS. The cells were lysed on ice with 200 μL of ice-cold immune-precipitation (IP) lysis buffer (10 mL of Pierce IP lysis buffer (87788, Thermo Scientific) supplemented with one Complete Ultra mini tablet with ethylenediaminetetraacetic acid (EDTA) (0589297001, Roche, Pleasanton, CA, USA), 5 mM *N*-ethylmaleimide (E3876, Sigma-Aldrich, St. Louis, MO, USA), and 1X phenylmethylsulfonyl fluoride (PMSF) immediately before use. The cells were then scrapped and collected into microtubes and spun at 13,000 rpm at 4 °C for 20 min. The supernatants were collected and kept on ice.

To prepare Dynabead-protein G (10007D, Life Technologies), the beads were mixed well and pipetted into a microfuge tube (50 μL for each IP sample). The beads were placed on a magnet to remove the buffer and then incubated with anti-HER2 antibody (clone 3B5, Millipore) diluted in the antibody binding and washing buffer provided. The beads and the antibody were incubated on a rotator at room temperature for 1 h. After the incubation, the beads were divided into individual microfuge tubes for each immunoprecipitation, and the buffer in each tube was removed while capturing the beads with a magnet. The cell lysate supernatants were then added to the beads and incubated on a rotator at 4 °C overnight. On the next day, the samples were allowed to continue incubation on a rotator at room temperature for 30 min before three washes with washing buffer. After the final wash, the beads were incubated with boiling 2X Laemmli buffer (161-0737, Bio-Rad, Hercules, CA, USA) and incubated on a 95 °C heat block for 5 min. The samples were then either stored at −80 °C or cooled down to room temperature for SDS-PAGE.

Protein samples were loaded in precasted Criterion TGX gels (567-1024, 567-1025, Bio-Rad) and ran in Tris/Glycine/SDS buffer (161-0732, Bio-Rad) diluted at 1X with Milli-Q water at 200 V constant voltage. The proteins were transferred to Immuno-Blot polyvinylidene difluoride (PVDF) membrane (162-0238, Bio-Rad) with Tris/Glycine buffer (161-0734, Bio-Rad) diluted at 1X and supplemented with 20% methanol using a standard transfer tank (HE62, Hoefer, Holliston, MA, USA) for 1 h in the cold room. The membranes were then rinsed and blocked with StartingBlock T20 blocking buffer (37543, Thermo Scientific, Waltham, MA, USA) at room temperature for 30 min before incubating with primary antibodies at 4 °C overnight. On the next day, the membranes were washed 3 × 15 min with TBST (1X Tris Buffered Saline (TBS) supplemented with 1% Tween-20) before incubation of horseradish peroxidase (HRP)-conjugated secondary antibodies (715-035-150, 711-035-152, Jackson Immuno Research, West Grove, PA, USA) on a rocker at room temperature for 1 h. After 3 × 15 min washes with TBST, the membranes were incubated with Amersham ECL prime or ECL select reagents (GE Healthcare Life Sciences, Chicago, IL, USA) on a rocker at room temperature for 5 min in the dark. The membranes were then imaged with ImageQuant LAS 4000 mini (GE Healthcare Life Sciences, Chicago, IL, USA). The band intensities were quantified by the ImageQuant IQ software. For purified exosomes, the protein concentrations of samples were determined by Nanodrop using A280. The samples were then normalized and loaded onto gels for SDS-PAGE and Western blotting.

### 2.9. Detection of Total HER2 ECD in Conditioned Media by ELISA

Total HER2 ectodomain (ECD) present in the conditioned media was detected and quantified by an ELISA sandwich assay. A standard-bind Meso Scale Discovery (MSD) 96-well assay plate (Meso Scale Discovery, Rockville, MD, USA) was coated with capture anti-human HER2 ECD antibody (Clone R002, Cat# 10004-R002, Sino Biological Inc, Chesterbrook, PA, USA) overnight at 4 °C. On the next day, the plate was washed with 1X ELISA wash buffer. It was then incubated with I-block buffer for 2 h at room temperature. After that, the plate was washed again with 1X ELISA buffer and incubated with the sample on a shaker for 45 min at room temperature. After another wash with 1X ELISA buffer, the plate was incubated with biotinylated anti-human HER2 ECD antibody (Clone 511, Cat# 10004-511, Sino Biological Inc.) diluted in heterophilic blocking reagent (HBR, Scantibodies, Santee, CA, USA) on a shaker for 45 min at room temperature. The plate was then washed with 1X ELISA buffer and incubated with streptavidin-sulfo-TAG diluted in I-block buffer on a shaker for 45 min at room temperature. After that, the plate was washed with 1X ELISA buffer and incubated with 1X Read Buffer T (Cat# R92TC-1, Meso Scale Discovery, Rockville, MD, USA). The plate was then transferred to MSD Sector Imager 6000 (Meso Scale Discovery, Rockville, MD, USA) for analysis within 10 min.

### 2.10. Exosome Purification by Ultracentrifugation

Conditioned media-derived exosomes were purified by ultracentrifugation as previously reported [44]. About 70 mL each of the conditioned media was filtered through a 0.2 μm filter unit before ultracentrifugation. The filtered conditioned media were kept on ice for later steps. They were centrifuged at 10,000× *g* for 30 min at 4 °C using an Optima L-80 XP ultracentrifuge and an SW 32 Ti rotor (Beckman Coulter, Carlsbad, CA, USA). The supernatant was transferred to a new tube and centrifuged at 100,000× *g* for 70 min at 4 °C. The pellet was washed with 1 mL PBS, and the fractions from other tubes were pooled together. The tube was then topped up to approximately 38 mL with PBS. The samples were then centrifuged again at 100,000× *g* for 70 min at 4 °C. The supernatant was decanted, and the pellet was resuspended in 40 μL of PBS and stored at −80 °C.

### 2.11. Quantification of Exosome Count by NanoSight

Purified exosome samples were thawed on ice and then diluted at 1:100 with PBS. The diluted samples were injected into a NanoSight for particle analysis. Each sample was analyzed six times, and the average of particle numbers per mL was calculated.

## 3. Results

### 3.1. Anti-HER2-Bs Induces Rapid HER2 Internalization Compared to Its Parental Arm Antibodies

**Figure 1:**
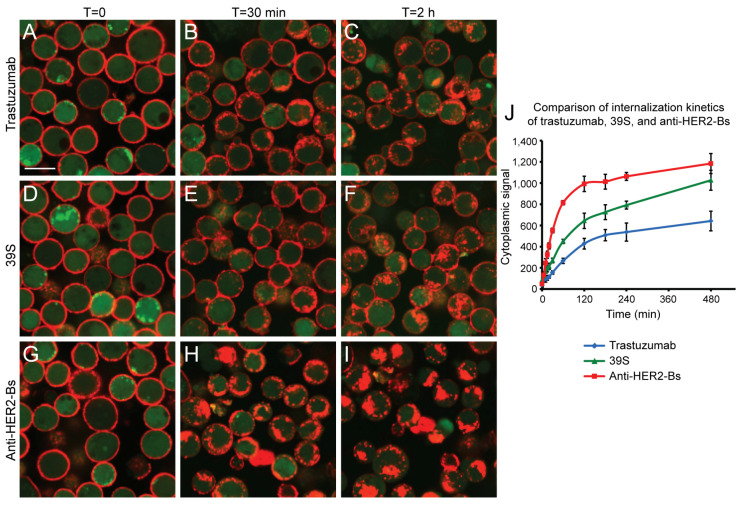
Rapid internalization of anti-HER2-Bs compared with trastuzumab or 39S in BT-474 cells. Internalization time courses of trastuzumab-AF647 (**A**–**C**), 39S-AF647 (**D**–**F**), and anti-HER2-Bs-AF647 (**G**–**I**) in cells stained with cytoplasmic dye, CFSE) (green), are shown. Before the initiation of internalization, fluorescence antibodies are localized on the cell surface (**A**,**D**,**E**). At 30 min, both trastuzumab-AF647 (red) and 39S-AF647 (red) are slowly internalized into the cells (**B**,**E**), while a majority of anti-HER2-Bs-AF647 (red) are already internalized (**H**). At 2 h, the surface-bound anti-HER2-Bs-AF647 is completely internalized (**I**), while both trastuzumab-AF647 and 39S-AF647 remain on the cell surface (**C**,**F**). (**J**) Antibody internalization time course was quantified by an image analysis algorithm. Data are plotted as mean ± standard deviation from 6 wells. Scale bar is 10 μm.

Anti-HER2-Bs comprises a human anti-HER2 monoclonal antibody, 39S-IgG1k, with two scFv domains of trastuzumab fused to the N-terminus of its heavy chain [43]. The biparatopic tetravalent antibody induced rapid internalization in various breast cancer cell lines [42]. To study the internalization kinetics of the molecule and compare it to that of its parental arm antibodies, BT-474 cells were bound with anti-HER2-Bs-AF647, trastuzumab-AF647, or 39S-AF647 and subjected to live cell imaging and quantitative analysis. Prior to the initiation of internalization, all three antibodies were bound to HER2 receptors on the cell surface (Figure 1A,D,G; shown in red). After 30 min, both trastuzumab-AF647 and 39S-AF647 showed limited internalization into the cells (Figure 1B,E), being present predominantly on the cell surface with a few internalized spots (red spots colocalized with cytoplasm dye CFSE), indicating slow internalization. In contrast, the majority of anti-HER2-Bs-AF647 was internalized in the same timeframe (Figure 1H). By 2 h, anti-HER2-Bs-AF647 was completely internalized and cleared from the cell surface (Figure 1I), while trastuzumab-AF647 and 39S-AF647 partially remained on the cell surface (Figure 1C,F). The time course of antibody-mediated HER2 internalization was analyzed using a quantitative algorithm (Figure 1J). Both trastuzumab-AF647 and 39S-AF647 antibodies were slowly internalized with an internalization half-life (t½) of approximately 120 min, whereas anti-HER2-Bs-AF647 induced rapid HER2 internalization with a t½ of about 30 min (Figure 1J). This result suggests a synergy (based on half-life) between the two binding epitopes in anti-HER2-Bs to induce rapid HER2 endocytosis. A similar trend of enhanced internalization of anti-HER2-Bs versus parental arm antibodies was observed in low HER2-expressing MCF-7 cells (Figure S2).

### 3.2. Anti-HER2-Bs-Induced Rapid Internalization Does Not Involve an Additional Endocytic Pathway

**Figure 2:**
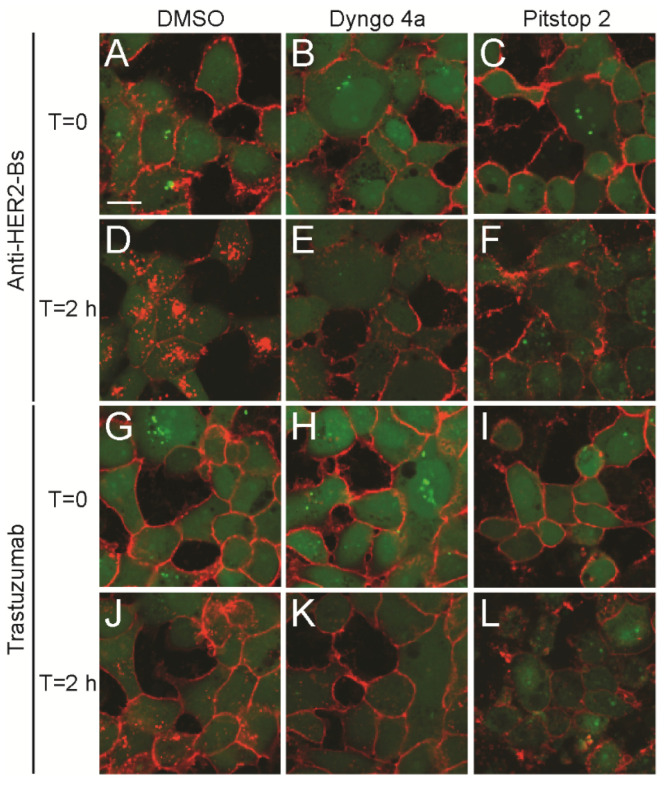
Effect of dynamin and clathrin inhibitors on anti-HER2-Bs and trastuzumab internalization. Overlays of anti-HER2-Bs-AF647 (**A**–**F**) and trastuzumab-AF647 (**G**–**L**) with the cytoplasm dye (green) in BT-474 cells treated with either DMSO (**A**,**D**,**G**,**J**), dynamin inhibitor Dyngo 4a (**B**,**E**,**H**,**K**), or clathrin light chain inhibitor Pitstop 2 (**C**,**F**,**I**,**L**) are shown. At T = 0, anti-HER2-Bs-AF647 (**A**,**B**,**C**) or trastuzumab-AF647 (**G**,**H**,**I**) was bound on the surface of the treated cells. At 2 h, the internalization of both anti-HER2-Bs-AF647 (red) and trastuzumab-AF647 (red) was inhibited in the presence of Dyngo 4a (compare **B**, **E** to **A**, **D** for anti-HER2-Bs; compare **H**, **K** to **G**, **J** for trastuzumab) or Pitstop 2 (compare **C**, **F** to **A**, **D** for anti-HER2-Bs; compare **I**, **L** to **G**, **J** for trastuzumab). Scale bar is 10 μm.

To test whether the anti-HER2-Bs-induced rapid internalization was due to the utilization of several entry pathways, small molecule inhibitors were used to disrupt dynamin- and clathrin-mediated endocytosis. The internalization of both anti-HER2-Bs-AF647 and trastuzumab-AF647 was inhibited in the presence of a highly potent dynamin I/II inhibitor, Dyngo 4a (compare Figure 2E to Figure 2D and Figure 2K to Figure 2J). In either control (DMSO) or Dyngo 4a-treated cells, fluorescent antibodies were localized on the cell surface before the initiation of internalization (Figure 2A,B,G,H). After two hours of incubation at 37 °C, anti-HER2-Bs-AF647 (Figure 2D) or trastuzumab-AF647 (Figure 2J) was internalized rapidly (Figure 2D) or slowly (Figure 2J) into the control cells, respectively. However, the internalization of both antibodies was significantly inhibited in the presence of the dynamin inhibitor. These results suggest that both anti-HER2-Bs and trastuzumab are internalized via dynamin-dependent endocytic pathways. To determine whether anti-HER2-Bs was internalized via clathrin-mediated endocytosis or utilized another endocytic pathway, we blocked clathrin-mediated endocytosis with a specific inhibitor against the clathrin light chain, Pitstop 2. Figure 2 shows that internalization of both anti-HER2-Bs and trastuzumab was efficiently inhibited by the clathrin inhibitor (compare Figure 2F to Figure 2D and Figure 2L to Figure 2J). These results indicate that both antibodies are internalized predominantly via clathrin-mediated endocytosis. Therefore, the rapid internalization of anti-HER2-Bs is not due to entry via additional endocytic pathways.

### 3.3. Concurrent Engagement of both Epitopes Is Necessary for the Anti-HER2-Bs-Induced Rapid Internalization

**Figure 3:**
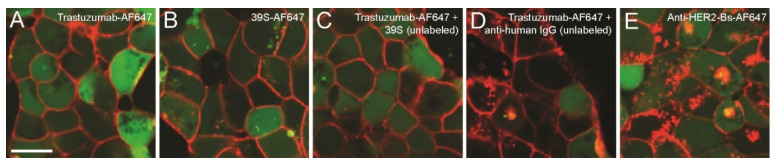
The combination of antibodies did not accelerate internalization. However, the addition of a secondary antibody accelerated trastuzumab internalization. Combined treatment with trastuzumab-AF647 (red) and unlabeled 39S (**C**) did not accelerate internalization of trastuzumab compared to trastuzumab-AF647 alone at 1 h (**A**). The internalization rate of trastuzumab-AF647 in the presence of 39S (**C**) is similar to the single antibody treatment with trastuzumab-AF647 (**A**) or 39S-AF647 (red) (**B**). By contrast, and similar to the previous figures, anti-HER2-Bs-AF647 (red) internalized rapidly into the cells (**E**). (**D**) Addition of anti-human IgG accelerated the internalization of trastuzumab compared to the untreated cells (compare **D** to **A**). However, the internalization rate of anti-HER2-Bs is still much more pronounced compared to the trastuzumab-AF647/anti-human IgG-treated cells (compare **E** to **D**). Cytoplasm dye, CFSE, is shown in green. Scale bar is 10 μm.

To test whether the rapid anti-HER2-Bs internalization is a result of the combination of trastuzumab and 39S epitopes in HER2 binding, cells were treated with both antibodies to determine if the combination of two antibodies can accelerate internalization. The internalization of combined treatment with trastuzumab and 39S was similar to either of them alone (compare Figure 3C to Figure 3A,B). By contrast, the internalization of anti-HER2-Bs was significantly faster (Figure 3E). These results suggest that the combination treatment of HER2 epitopes with two antibodies did not effectively accelerate internalization.

Dimerization or crosslinking of surface receptors by bivalent antibody binding has been known to induce receptor internalization. To test if antibody crosslinking on the cell surface may be the cause of rapid anti-HER2-Bs internalization, we examined the internalization of trastuzumab in a crosslinking-induced condition. A secondary antibody, polyclonal anti-human IgG, was added to BT474 cells prebound with trastuzumab-AF647. Polyclonal anti-human IgG binds to Fc regions of trastuzumab and can crosslink adjacent antibody-receptor complexes on the cell surface. As shown in Figure 3D, the presence of the secondary anti-human IgG increased the internalization of trastuzumab-AF647 when compared to trastuzumab-AF647 alone. The increase in internalization, however, was much lower compared to the rapid internalization induced by anti-HER2-Bs (Figure 3E). These results suggest that cell surface receptor crosslinking may only partially account for accelerated anti-HER2-Bs internalization.

Since neither crosslinking of the parental arm antibodies nor the combination of two parental arms from separate molecules induced rapid internalization comparable to anti-HER2-Bs, we hypothesized that concurrent binding of both trastuzumab and 39S epitopes in a bispecific molecule could play a critical role in rapid internalization of anti-HER2-Bs. To test this hypothesis, we examined anti-HER2-Bs-AF647 internalization under conditions where one of the parental arm epitopes on HER2 was blocked by an excess of the parental arm antibody, thus forcing anti-HER2-Bs to bind only through the second epitope. Specifically, cells were blocked with an excess of unlabeled trastuzumab at 4 °C to occupy all the binding epitopes at domain IV on HER2 receptors, followed by incubation with anti-HER2-Bs-AF647. In this scenario, anti-HER2-Bs-AF647 should bind to the HER2 receptors via the 39S-binding epitopes only and thus mimic the binding of the parental arm 39S antibody to the HER2 receptors. Trastuzumab and 39S were also included as controls.

**Figure 4:**
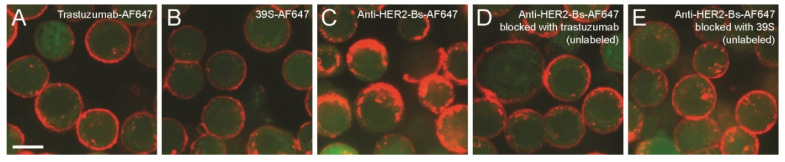
Blocking one epitope on HER2 receptors slowed down the internalization of anti-HER2-Bs-AF647 significantly. Cytoplasm dye, CFSE, is shown in green. Similar to the previous figures shown, anti-HER2-Bs-AF647 (red) internalized rapidly (**C**) compared to either trastuzumab-AF647 (red) (**A**) or 39S-AF647 (red) (**B**) at 2 h. (**D**) By contrast, blocking of trastuzumab-binding epitopes significantly slowed down the internalization of anti-HER2-Bs-AF647 at 2 h (compare **D** to **C**) and resembled the internalization of 39S alone (compare **D** to **B**). (**E**) Similarly, blocking of the other epitopes (39S-binding epitopes) also slowed down the anti-HER2-Bs-AF647 internalization (compare **E** to **C**). Note that at the start of internalization (T = 0), similar fluorescent signals (AF647) were observed for all tested conditions (**A**–**E**) (see Figure S3). Scale bar is 10 μm.

At the start of internalization (T = 0), trastuzumab-AF647, 39S-AF647, anti-HER2-Bs-AF647 as well as anti-HER2-Bs, blocked with either trastuzumab or 39S, showed similar fluorescent signals (Figure S3), indicating similar binding of each molecule, and that when one epitope on HER2 was blocked (by an unlabeled trastuzumab or an unlabeled 39S), anti-HER2-Bs was bound to the receptor via the other epitope. Blocking trastuzumab-binding epitopes slowed down the internalization of anti-HER2-Bs-AF647 effectively. Similar to the results presented in Figure 1 and Figure 3, anti-HER2-Bs exhibits rapid internalization compared to either trastuzumab or 39S antibody by 2 h (Figure 4A–C). By contrast, the internalization of the trastuzumab-blocked anti-HER2-Bs-AF647 was slowed down significantly compared to the control cells where the epitope was not blocked (compare Figure 4D to Figure 4C) and resembled the internalization pattern of the parental arm 39S antibody (compare Figure 4D to Figure 4B). In a similar manner, blockade of the 39S-binding epitope with an excess of the unlabeled 39S antibody resulted in decreased anti-HER2-Bs internalization (compare Figure 4E to Figure 4C). These results strongly suggest that concurrent binding of both epitopes is necessary for the anti-HER2-Bs-induced rapid internalization.

### 3.4. Anti-HER2-Bs Induces Efficient HER2 Degradation

**Figure 5:**
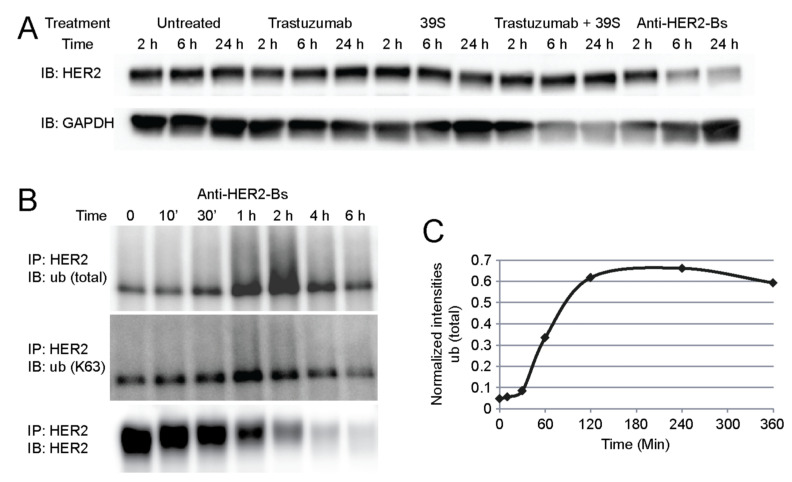
Anti-HER2-Bs, but not trastuzumab, 39S, or both in combination, induced efficient HER2 degradation. (**A**) The level of total HER2 was not changed in the cells treated with trastuzumab, 39S, or both in combination. However, treatment with anti-HER2-Bs resulted in a decrease in total HER2 expression by 6 h. (**B**) Anti-HER2-Bs induces HER2 receptor ubiquitination, and this coincides with HER2 degradation. Treatment with anti-HER2-Bs resulted in an increase in total HER2 ubiquitination at 1–2 h and K63-specific ubiquitination at 1 h. (**C**) Quantification of HER2 total ubiquitination level normalized to total HER2 receptor level. The normalized level of HER2 total ubiquitination increased from 30 min post treatment and was sustained during 2–6 h post treatment.

In addition to rapid HER2 internalization, anti-HER2-Bs also induced efficient HER2 receptor degradation. When SK-BR-3 cells were treated with anti-HER2-Bs, an efficient degradation of HER2 was already observed by 6 h. Treatment with either trastuzumab or 39S did not affect the level of total HER2 receptors throughout the time course (Figure 5A). The combined treatment of trastuzumab and 39S resulted in a modest degradation of HER2 receptors but it was not comparable to the efficient degradation induced by anti-HER2-Bs (Figure 5A).

Receptor ubiquitination is often involved in lysosomal trafficking and degradation. To determine if anti-HER2-Bs-induced degradation involves HER2 ubiquitination, we monitored HER2 ubiquitination and degradation in SK-BR-3 cells over a 6 h time course. Cells treated with anti-HER2-Bs for the indicated time periods were lysed, and HER2 was immunoprecipitated from total cell lysates using the anti-HER2 antibody. The anti-HER2 antibody (clone 3B5), which binds to the C-terminal domain of HER2, was selected for HER2 pull-down to avoid interference with anti-HER2-Bs bound on the N-terminus. Western blots of the HER2 immunoprecipitates probed for HER2, total ubiquitin, and K63-ubiquitin are shown in Figure 5B. The HER2 detection panel shows ~50% of receptors degraded by 1 h and a low level of HER2 after 2 h under anti-HER2-Bs treatment. Degradation of HER2 parallels with an increase in ubiquitination as detected by an anti-total ubiquitin antibody, P4D1 (top panel, Figure 5B). When the amount of HER2 ubiquitination was normalized to total HER2 level to account for reduced HER2 amounts (Figure 5C), HER2 ubiquitination was observed at 30 min, reached the maximal level at 2 h, coinciding with HER2 degradation, and was sustained throughout the 6 h time course (Figure 5C). The same samples were probed with anti-ubiquitin K63 chain-specific antibody to detect K63 polyubiquitination. Figure 5B (middle panel) shows a time-dependent increase in K63-specific ubiquitination. Since K63 ubiquitination is known to signal protein for multivesicular bodies (MVBs)/lysosomal trafficking, this result suggests that anti-HER2-Bs routed the receptor to the lysosomal pathway.

### 3.5. Antibodies Mediate Distinct Trafficking Pathways and Fates of HER2 Receptors

#### 3.5.1. Internalized Anti-HER2-Bs Stayed Associated with HER2 while Internalized Trastuzumab and 39S Dissociated from HER2

Treatment with anti-HER2-Bs and parental arm antibodies resulted in differential fates of HER2 receptors. To dissect the intracellular trafficking of the internalized antibody-HER2 complexes, we independently followed antibodies and HER2 using immunofluorescence microscopy. BT-474 cells were prebound with anti-HER2-Bs-AF647, trastuzumab-AF647, and 39S-AF647 and allowed to internalize for 30 min, 2 h, and 8 h. The cells were fixed and stained using AF488-labeled anti-HER2 antibodies after permeabilization. To avoid competition with anti-HER2-Bs, trastuzumab, or 39S, we detected HER2 receptors with an antibody against the C-terminal domain of HER2.

**Figure 6:**
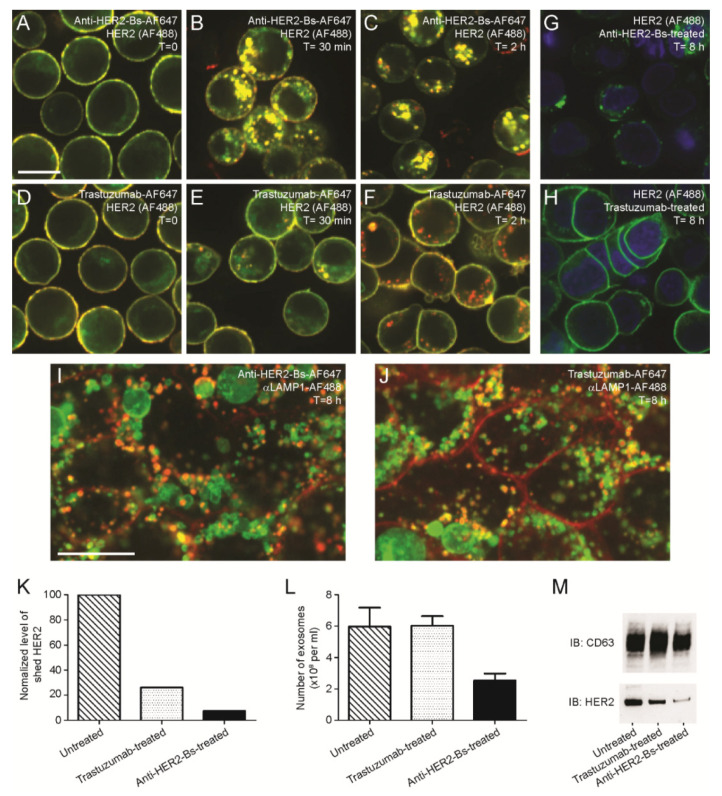
Anti-HER2-Bs and trastuzumab mediated distinct intracellular trafficking of internalized HER2 receptors. Internalization time courses of anti-HER2-Bs-AF647 (**A**–**C**) and trastuzumab-AF647 (**D**–**F**) are shown. (**A**,**D**) Before the initiation of internalization, both anti-HER2-Bs-AF647 and trastuzumab-AF647 (red) were colocalized with HER2 receptors on the cell surface (green) and appeared yellow. (**B**,**E**) At 30 min, the antibody-HER2 complexes (appearing as yellow puncta) were internalized into the cells. Consistent with the previous figures, the internalization of anti-HER2-Bs-AF647 is more rapid compared to trastuzumab-AF647 (compare **D** to **G**). (**C**,**F**) Anti-HER2-Bs-AF647 and trastuzumab-AF647 mediated strikingly different trafficking of HER2 receptors at 2 h. (**C**) Internalized anti-HER2-Bs-AF647 (red) remained colocalized with HER2 receptors (green) and appeared as yellow puncta. However, trastuzumab-AF647 was dissociated from HER2 receptors and appeared as red puncta in the cells (**F**). (**G**,**H**) Treatment with anti-HER2-Bs, but not trastuzumab, resulted in complete surface clearance of HER2 receptors at 8 h. (**I**,**J**) Internalized anti-HER2-Bs-AF647 and trastuzumab-AF647 are colocalized with a lysosomal marker, LAMP1-AF488, at 8 h. The puncta of internalized anti-HER2-Bs-AF647 (**I**) or trastuzumab-AF647 (**J**) (red), colocalized with the LAMP1-AF488-positive compartments (green) merging in yellow. Treatment with anti-HER2-Bs resulted in the complete clearance of the antibody from the cell surface by 8 h (**I**). By contrast, there was a significant amount of trastuzumab remaining on the cell surface (**J**). Scale bars are 10 μm. (**K**) Treatment with anti-HER2-Bs resulted in a ~93% reduction of HER2 ectodomain (ECD) shedding in the conditioned culture media compared to the untreated cells. Treatment with trastuzumab also reduced HER2 ECD shedding by ~76%. (**L**,**M**) In addition, treatment with anti-HER2-Bs also reduced the number of exosome particles and the level of HER2 expression in the normalized exosomal fraction. CD63 is the protein marker for exosomes purified from conditioned culture media.

Distinct trafficking HER2 pathways were observed after treatment of cells with anti-HER2-Bs versus trastuzumab or 39S. Both trastuzumab and 39S showed similar results (see Supplementary Materials, Figure S4). As such, a comparison of anti-HER2-Bs and only trastuzumab is presented in Figure 6. Internalized anti-HER2-Bs was found to be colocalized with HER2 receptors (Figure 6A–C), while the internalized trastuzumab dissociated from HER2 at 1–2 h (Figure 6D–F). Before the initiation of internalization (T = 0), both antibodies and HER2 were colocalized on the cell surface (Figure 6A,D). After 30 min, the anti-HER2-Bs-HER2 complex was internalized rapidly into the cells (Figure 6B), while the trastuzumab-HER2 complex was slowly internalized (Figure 6E). At 2 h, the internalized anti-HER2-Bs-HER2 complexes stayed associated in the cell (Figure 6C), but the internalized trastuzumab was found to be dissociated from HER2 (Figure 6F). Treatment with anti-HER2-Bs resulted in degradation of total HER2 expression level (Figure 5) and loss of surface expression by 8 h (Figure 6G). Internalized trastuzumab dissociated from HER2, allowing a constant level of HER2 receptors to be maintained on the cell surface (Figure 6H). Our data collectively suggest that internalized HER2 is recycled back to the cell surface while the internalized trastuzumab is degraded in lysosomes. Internalized anti-HER2-Bs-HER2 complexes stay associated and are trafficked to lysosomes for degradation.

To determine the fate of internalized antibodies, we examined anti-HER2-Bs2-AF647 and trastuzmab-AF647 at the later time point to determine if antibodies were routed to the lysosomes. After 8 h of internalization, both internalized anti-HER2-Bs-AF647 and trastuzumab-AF647 were colocalized with the lysosomal protein marker LAMP1 (shown in yellow as merged red and green signals, Figure 6I,J). Since the extent of trastuzumab internalization was less pronounced than for anti-HER2-Bs (Figure 1C,I, Figure 3A,E and Figure 6C,F), there was still a significant amount of trastuzumab-AF647 on the cell surface at 8 h (red). The majority of surface-bound anti-HER2-Bs was internalized and cleared from the cell surface (Figure 6G). These results indicate that the internalized anti-HER2-Bs and trastuzumab are trafficked to lysosomes.

As shown in Figure S4, 39S exerted similar effects to trastuzumab: slow internalization, separation from HER2 receptors in ~2 h and, at a later time point (8 h), trafficking to lysosomes. Upon dissociation form 39S, HER2 potentially recycled back, as the HER2 levels at the cell surface were maintained over the time of antibody treatment. In good agreement are the results shown in Figure 5A, where 24 h incubation with 39S did not induce degradation of total HER2. Collectively, the results of trastuzumab and 39S demonstrated that rapid internalization and redirected trafficking of anti-HER2 was due to both paratopes engineered in the same molecule.

#### 3.5.2. Anti-HER2-Bs Reduces the Amount of HER2 Ectodomain Shedding

HER2 receptors on the cell surface are constantly undergoing proteolytic cleavage of the ECD [45]. Profound HER2 degradation upon anti-HER2-Bs continuous treatment resulted in reduced HER2 surface expression (Figure 6G) and may therefore reduce levels of shed HER2. To examine levels of HER2 ECD, we utilized an ELISA-based immunoassay. The assay measures total HER2 ECD, bound and unbound to either anti-HER2-Bs or trastuzumab. Figure 6K shows measurement of HER2 ECD in the conditioned media of SK-BR-3 cells, untreated or treated with either of the antibodies. Consistent with the degradation route of the internalized HER2 receptors, anti-HER2-Bs significantly reduced the level of HER2 ECD by ~93% compared to the untreated cells (Figure 6K). Treatment with trastuzumab is known to inhibit HER2 shedding because it binds to domain IV and blocks the cleavage site of the receptor [46]. Consistently, we showed that trastuzumab treatment resulted in a ~76% reduction of HER2 ECD shedding. Since anti-HER2-Bs contains the HER2 binding domain of trastuzumab, a similar level of inhibition of HER2 ECD shedding was anticipated. However, anti-HER2-Bs inhibited HER2 shedding to a greater extent than trastuzumab (~93% vs. ~76% inhibition), indicating the presence of an additional mechanism. We believe that this additional mechanism was rapid internalization and degradation of HER2 diminishing the levels of receptor available for shedding on the cell surface.

#### 3.5.3. Anti-HER2-Bs Reduces the amount of HER2 in Exosomes

Internalization of plasma membrane portions into endosomes and subsequent internalization of endosomal membrane into smaller vesicles—MVBs—is a part of the normal membrane turnover and endocytic trafficking. Once HER2 is routed through the endocytic pathway to MVBs, it can be either routed to a degradation pathway by MVB fusion with lysosomes or it can be released as cell-derived vesicles (exosomes) by fusion of MVBs with plasma membrane. To study the effect of anti-HER2-Bs-induced HER2 degradation on exosomal transport, SK-BR-3 cells, known to constitutively release HER2 exosomes [47], were treated with anti-HER2-Bs or trastuzumab and compared with untreated cells. Exosomes were purified from the conditioned media using ultracentrifugation [44]. Our results showed that the HER2 loading into MVBs, as seen from CD63, a marker for MVB, and the release of HER2-positive exosomes as determined by exosome enumeration using NanoSight, was not affected by trastuzumab. In contrast, cells treated with anti-HER2-Bs had significantly reduced numbers of HER2 exosomes and HER2 expression (Figure 6L,M).

## 4. Discussion

Targeting two non-overlapping epitopes of HER2 using a combination of antibodies or a bispecific molecule induced accelerated internalization and degradation of HER2 receptors [34,36,37,41] as well as enhanced anti-tumor-inhibitory activity [37,41,48,49]. However, the combination of antibodies with non-overlapping epitopes does not necessarily result in accelerated target-mediated internalization. For example, in our study, the combination of antibodies targeting two non-overlapping epitopes, a trastuzumab-targeted epitope in domain IV and a 39S-targeted epitope in domain II, did not accelerate internalization (Figure 3C). This suggests that the ability of two monoclonal antibodies to synergize in such combination treatments is epitope-dependent and not universal. Although the two epitopes did not synergize in the combinational treatment, engineering both epitope-binding regions into a single bispecific molecule significantly enhanced internalization. This enhanced internalization effect could be a result of an increased ability of anti-HER2-Bs to crosslink the receptor. To support this hypothesis, analysis of binding interactions between the recombinant extracellular domain of HER2 (ECD) and anti-HER2-Bs or trastuzumab using HPLC SEC-MALS (size exclusion chromatography-multiangle light scattering) showed that anti-HER2-Bs was indeed able to form high molecular weight complexes with HER2 ECD, whereas trastuzumab was not [42]. We believe that the formation of high molecular complexes is due to the ability of anti-HER2 to crosslink receptors through multi-epitope interactions, while trastuzumab can bind to HER2 ECD via simple bivalent antibody–receptor interaction.

It has been previously hypothesized that the formation of large receptor-antibody complexes or lattices on the cell surface is responsible for the efficient internalization [36,37]. Anti-HER2-Bs is a tetravalent bispecific antibody and contains two trastuzumab and two 39S epitope-binding regions on an IgG backbone. This design confers its multivalent-binding ability to bind to, and crosslink, multiple HER2 receptors more efficiently. Anti-HER2-Bs also has a higher affinity to the HER2 receptors compared to trastuzumab or 39S [50,51], and may result in the higher avidity from the receptors crosslinking. Importantly, we showed that the addition of a secondary anti-human antibody was able to accelerate the internalization of trastuzumab (Figure 3D). This suggests that a higher order binding or crosslinking of the antibody on the cell surface could accelerate internalization. In a recent computational simulation study, simultaneous epitopes binding has been suggested to play an important role in maximizing target binding and inhibition [52]. We demonstrated that the anti-HER2-Bs-mediated rapid internalization can be slowed down effectively by preoccupying one of the binding epitopes on the HER2 receptors (Figure 4). These results strongly suggest concurrent epitopes binding is necessary for inducing synergy in the rapid internalization.

**Figure 7:**
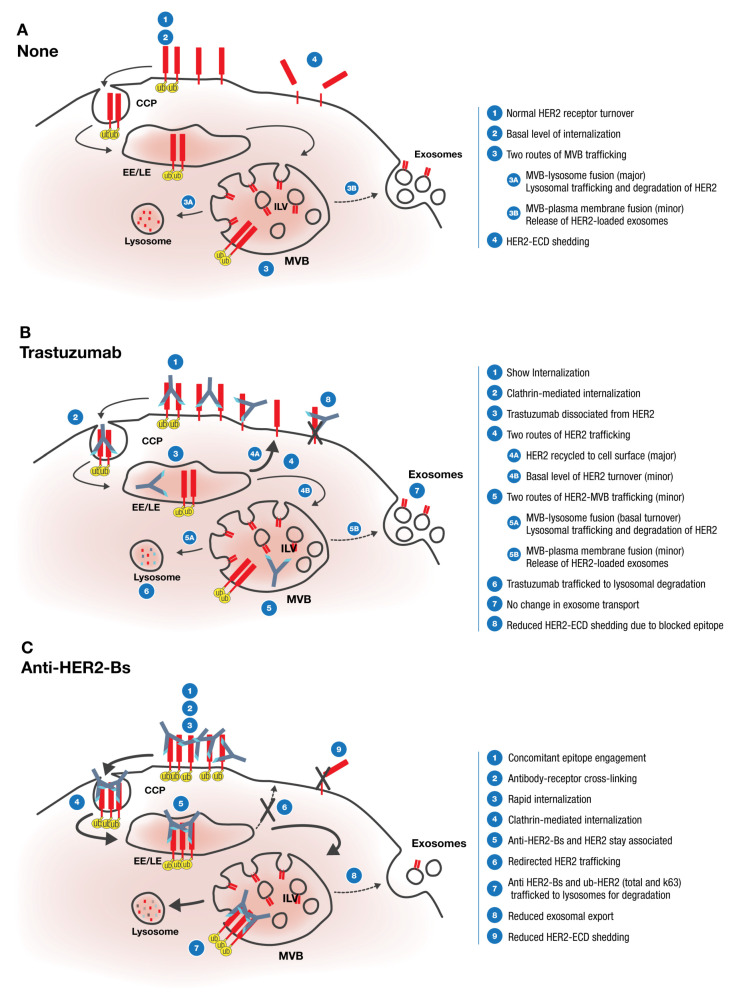
A proposed model of anti-HER2-Bs- and trastuzumab-mediated internalization, trafficking, and the fate of HER2 receptors. Please note that the receptors are drawn unproportionally to highlight the crosslinking on the cell surface and the intracellular trafficking. (**A**) In the untreated cells, the HER2 receptors are predominately localized on the cell surface. As part of the normal turnover process, a basal level of HER2 continuously undergoes internalization and catabolism. The pool of cell surface HER2 is replenished by the de novo synthesis of HER2 receptors. The internalized HER2 is then trafficked to multivesicular bodies (MVBs) for subsequent sorting. The HER2-loaded MVBs can fuse either with lysosomes for degradation, or with plasma membrane to release intra-luminal vesicles (ILVs) as exosomes. In addition, HER2-ECDs are constantly shedding from the receptors on the cell surface. (**B**) Treatment with trastuzumab resulted in a slow internalization of HER2 receptors via a clathrin-mediated pathway. The internalized trastuzumab-HER2 complexes are dissociated in the endosomes. The dissociated HER2 receptors are then recycled back to the cell surface. Similar to the untreated cells, a basal level of HER2 is internalized and trafficked to MVBs as a process of normal turnover, leading to either degradation of HER2 or release of HER2-loaded exosomes. On the other hand, the dissociated trastuzumab is trafficked to MVBs and lysosomes for degradation. Treatment with trastuzumab did not affect the level of exosome formation. However, binding of trastuzumab to the domain IV of the HER2 receptors on the cell surface inhibits the cleavage of HER2-ECD. (**C**) Concurrent engagement of HER2 epitopes by the bivalent bispecific anti-HER2-Bs antibody may induce crosslinking of HER2 receptors to form high molecular weight complexes on the cell surface. The anti-HER2-Bs-HER2 complexes are internalized rapidly via a clathrin-mediated pathway. The anti-HER2-Bs-HER2 complexes stay associated and traffic through the endocytic pathway. Instead of being recycled back to the cell surface, anti-HER2-Bs induces redirected trafficking of HER2 receptors to lysosomes for degradation. The degradation of HER2 receptors coincides with an increase in HER2 ubiquitination (total and K63). As a result of the lysosomal targeting, the anti-HER2-Bs-HER2-loaded multivesicular bodies are not able to fuse with the cell membrane, and thus this results in reduced exosomal export. In addition, rapid internalization of surface anti-HER2-Bs-HER2 complexes results in a fast clearance of surface HER2 receptors, thus leading to a reduction of HER2 ECD shedding on the cell surface.

Anti-HER2-Bs not only induced rapid internalization of the receptor but also modulated its intracellular trafficking. Figure 7 shows a proposed model for antibody modulation of HER2 internalization, trafficking, and ultimate fate. In untreated cells, HER2 undergoes normal receptor turnover including internalization, catabolism, de novo synthesis, and EDC shedding (Figure 7A). Internalized HER2 traffics through the endocytic pathway to MVBs, where it can be either routed for degradation by MVB fusion with lysosomes or it can be released as cell-derived vesicles (exosomes) by fusion of MVBs with plasma membrane. Trastuzumab treatment induces slow internalization of HER2 with dissociation of the antibody-receptor complex in endosomes in ~2 h and subsequent release of HER2 for recycling to the cell surface. As such, trastuzumab does not significantly alter HER2 intracellular trafficking from its normal turnover. Indeed, our study showed that trastuzumab neither induced receptor degradation nor altered exosomal release (Figure 7B). The HER2-ECD shedding was reduced in trastuzumab-treated cells. This, however, was due to trastuzumab binding to HER2 at the site, overlapping with the ECD cleavage domain, and resulting in inhibition of shedding. In contrast to trastuzumab, anti-HER2-Bs, with its ability to crosslink multiple receptors and generate high molecular weight complexes, induces fast HER2 internalization. Because of high affinity interactions, anti-HER2-Bs stays associated with HER2 through the entire endocytic pathway, preventing HER2 recycling and eventually trafficking receptors to lysosomes for degradation (Figure 7C). Our study showed that the anti-HER2-Bs-induced degradation of HER2 receptors paralleled with the HER2 ubiquitination (total and K63). K63 ubiquitination is a known marker of MVB/lysosomal trafficking, indicating that anti-HER2-Bs directed the receptor to the lysosomal pathway. Due to lysosomal targeting, the anti-HER2-Bs-HER2-loaded MVBs are not able to fuse with the cell membrane, resulting in inhibition of exosomal export, as observed in the present study. Rapid internalization of surface anti-HER2-Bs-HER2 removes HER2 receptors from the cell surface, resulting in a decrease in HER2-ECD shedding. 

In the proposed model, both trastuzumab and anti-HER2-Bs antibodies were trafficked to lysosomes for degradation (Figure 7B,C). The antibody catabolism suggested in the model corroborates the observed results of non-linear pharmacokinetic (PK) profiles for trastuzumab in a clinical study of patients with HER2-overexpressing metastatic breast cancers [40], as well as for MEDI4276 in a clinical study of patients with HER2+ advanced breast or gastric cancer [53].

Internalization kinetics of therapeutic antibodies is an important parameter in pharmacokinetic-pharmacodynamic (PK-PD) modeling [30,32,54], which can be used to evaluate potential drug targets, establish antibody engineering objectives, and predict clinical dose [55]. The internalization kinetics also provides important information for the design of a therapeutic antibody. Antibodies with a slower target-mediated internalization rate are generally preferred in ADCC or CDC formats as they can be maintained on the surface of target cells for a sufficient amount of time. Antibodies that induce rapid target-mediated internalization are more favorable in ADC development as they can rapidly deliver their cytotoxic payloads. However, it is also true that not every ADC antibody is fast internalizing. For example, T-DM1 (Kadcyla^®^) and T-DXd (Enhertu^®^) were developed based on the slowly internalizing trastuzumab [56]. By conjugating a cytotoxic payload to trastuzumab, Kadcyla displays ADCC-based cell killing as well as ADC-based toxin-mediated cell killing [56].

Rapid antibody internalization, which involves efficient crosslinking of surface receptors, would be dependent on receptor density on the cell surface to promote crosslinking. In cells with low surface expression of target receptors, the receptors are sparsely localized, and therefore crosslinking events might be limited, leading to formation of smaller or fewer complexes. In such a scenario, even a bivalent bispecific antibody, such as anti-HER2-Bs, will bind to sparse receptors in a manner similar to a conventional monospecific bivalent monoclonal antibody and internalize at the respective speed. We hypothesize that there are a critical receptor number and density threshold on the cell surface that trigger rapid internalization. In line with this hypothesis is slower internalization of anti-HER2-Bs in low HER2 MCF-7 cells (Figure S2) and other cancer cell lines with low HER2 expression [42]. HER2 is known to be expressed on heart tissue, posing a risk of cardiotoxicity for anti-HER2 ADCs; however, the expression level of HER2 is low. In good agreement with the slow internalization at low receptor density were the results of no cardiotoxicity in cynomolgus monkey toxicology studies of MEDI4276, an ADC version of anti-HER2-Bs [42].

Anti-HER2-Bs is a bivalent bispecific molecule. Designing a tetravalent bispecific antibody is challenging because the multivalent binding of an antibody could crosslink cell surface antigens and trigger undesirable outcomes such as formation of aggregates [57]. In addition, a very fast internalization rate can lead to rapid drug elimination and low exposure, which for ADC molecules may be translated into a low therapeutic index [58]. An alternative approach to avoid this challenge is to construct a bispecific molecule with monovalent antigen binding domains. Depending on the drug target and design of the therapeutic antibody, it is critical to consider whether a monovalent or bivalent bispecific antibody would be a better approach to confer clinical efficacy. Since rapid internalization and lysosomal trafficking of an antibody are critical factors for an ADC molecule, a bivalent bispecific format could be more advantageous, especially for ADCs targeting slowly internalizing receptors. An optimal selection of non-overlapping epitopes can then be engineered to induce accelerated internalization. The design of anti-HER2-Bs as a bivalent bispecific antibody, allowing concurrent binding of epitopes, triggered rapid internalization and efficient degradation, validating the utility of the approach in the ADC development. Collectively, the results of this study not only provide important information about the mechanism of action of the anti-HER2-Bs antibody and the biology of HER2 internalization, but also support the rational design of future anti-HER2 and ADC therapeutics.

## Supplementary Materials

The following are available online at https://www.mdpi.com/2073-4468/9/3/49/s1, Figure S1: Blocking of Fc-mediated antibody binding to HER2-expressing cells using FcR blocking reagent; Figure S2: Internalization of trastuzumab, 39S, and anti-HER2-Bs in low HER2-expressing MCF-7 cells; Figure S3: Blocking one epitope on HER2 receptors slowed down the internalization of anti-HER2-Bs-AF647 but did not reduce its cell binding; Figure S4: Anti-HER2-Bs and parental arm antibodies (trastuzumab and 39S) mediated distinct intracellular trafficking of internalized HER2 receptors.
