# Supplementary material for: Molecular Mechanism of HER2 Rapid Internalization and Redirected Trafficking Induced by Anti-HER2 Biparatopic Antibody
Source: Antibodies (Basel). 2020 Sep 18;9(3):49. doi: 10.3390/antib9030049 (PMC7551206; doi:10.3390/antib9030049)
Supplement: Supplementary file 1 [file antibodies-09-00049-s001.pdf]

antibodies

MDPI

Supplementary Materials

Molecular mechanism of HER2 rapid internalization and redirected trafficking induced by anti-HER2 biparatopic antibody

Jackie Cheng,[1†] Meina Liang[1], Miguel F. Carvalho[2‡], Natalie Tigue[2], Raffaella Faggioni[1§], Lorin K. Roskos[1§,] and Inna Vainshtein[1§*]

[1] Integrated Bioanalysis, Clinical Pharmacology and Safety Sciences, R&D, AstraZeneca, South San Francisco, CA, USA
[2] Antibody Discovery and Protein Engineering, R&D, AstraZeneca, Cambridge, UK
* Correspondence: ivainshtein@exelixis.com; inna.vainshtein@gmail.com

Current affiliations: [†]Precision For Medicine, Redwood City, CA, USA; [‡]InnovPlantProtect, Elvas, Portugal; [§]*Exelixis, Alameda, CA, USA*

Supplementary materials for the section 2.4 "Cell staining for antibody internalization"

The FcR blocking reagent efficiently blocks Fc receptors on the target antigen-expressing cells, inhibiting antibody binding via Fc-domain, so that the antibody would bind the cells via antigen. Figure S1 shows that pre-incubation of cells with FcR blocking reagent prior to antibody staining was sufficient to completely inhibit Fc-mediated binding of anti-HER2-Bs-AF647. No cell binding was observed in presence of IgG-AF647 indicating full blockade of Fc-mediated binding. The HER2-mediated binding is observed on the cell surface in the presence anti-HER2-Bs-AF647.

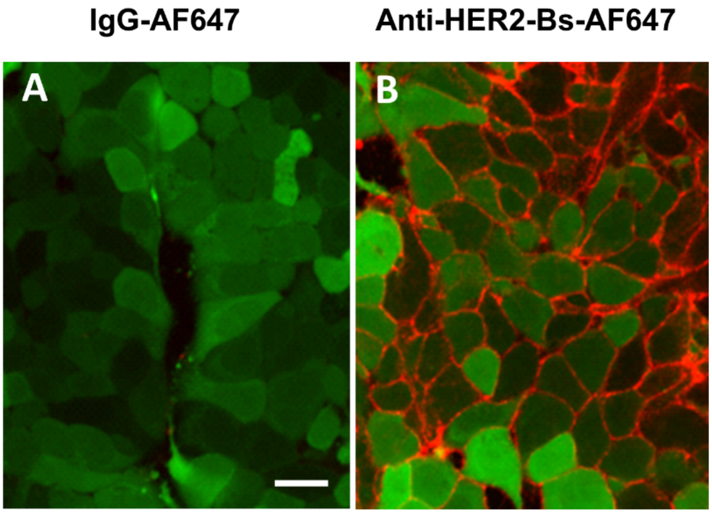

Figure S1. Blocking of Fc-mediated antibody binding to HER2-expressing cells using FcR blocking reagent. MCF-7 cells were pre-blocked with FcR blocking reagent and then stained with CFSE for cytoplasm (green) followed by incubation with 2 µg/mL of a control antibody, IgG-AF647 (A) or anti-HER2-Bs-AF647 (B). AF647 is shown in red. Scale bar is 10 µm.

Supplementary materials for the section 3.1 "Anti-HER2-Bs induces rapid HER2 internalization compared to its parental arm antibodies"

Comparative analysis of anti-HER2-Bs, trastuzumab and 39S in low HER2-expressing (~3,646 HER2 receptor/cell, [42]) MCF-7 cells is shown in Figure S1. At 2 h of internalization conditions anti-HER2-Bs demonstrated fully advanced internalization (I), whereas trastuzumab (C) or 39S (F) induced very limited

internalization. Overall, internalization in MCF-7 cells was not as rapid as in high-expressing BT-474 cells, corroborating the hypothesis (discussed in the discussion section) that critical receptor number is needed to trigger rapid internalization. Sparse HER2 receptors may not be efficiently cross-linked, even when the molecule has an ability to bind to these two non-overlapping epitopes on HER2.

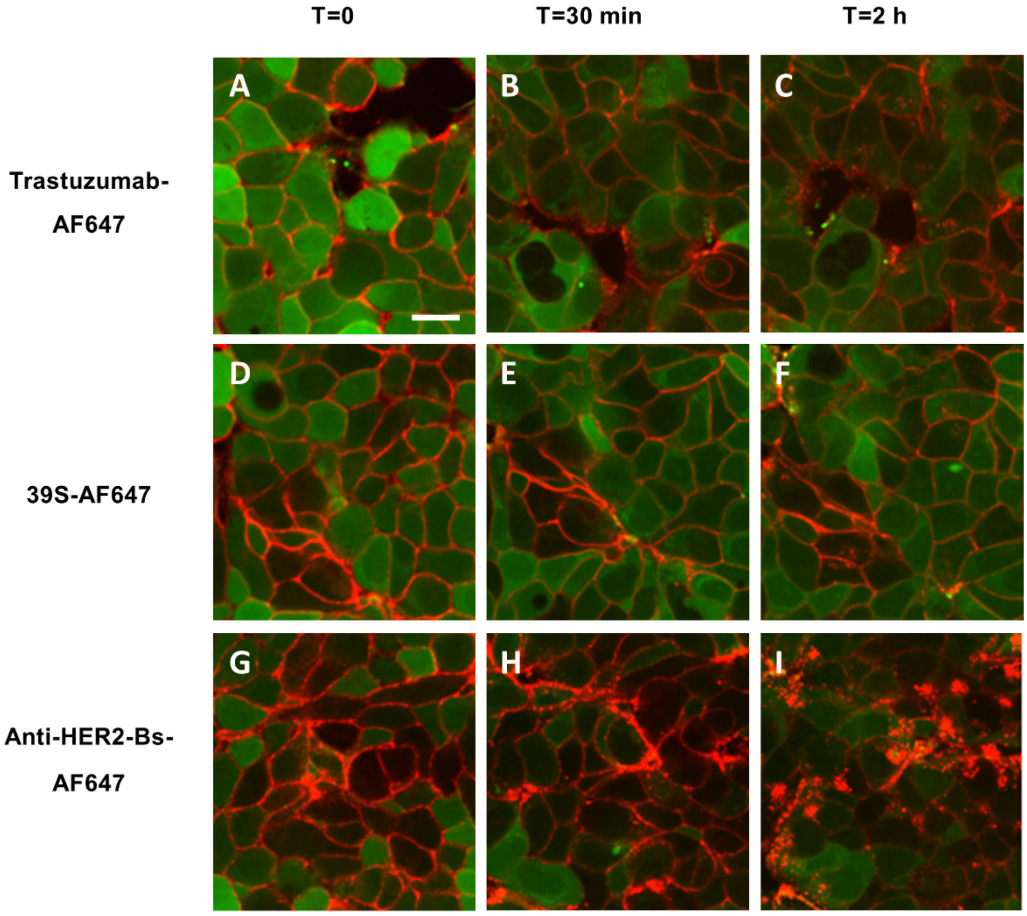

Figure S2. Internalization of trastuzumab, 39S and anti-HER2-Bs in low HER2-expressing MCF-7 cells. The experiments were performed as described in Material and Methods (sections 2.4 and 2.6). CFSE, a cytoplasm dye is shown in green. An anti-HER2-Bs showed an enhanced internalization (I) if compared with trastuzumab (C) or 39S (F). Overall anti-HER2-Bs internalization in MCF-7 cells was much slower than in BT474 cells (2 h time point (Figure 1I). While anti-HER2-Bs had fully advanced internalization at 30 min in BT-474 cells (Figure 1H), in MCF-7 cells the internalization had only started (H) at this time. Scale bar is 10 µm.

Supplementary materials to Figure 4 (section 3.3 "Concurrent engagement of both epitopes is necessary for the anti-HER2-Bs-induced rapid internalization")

Figure S3 includes "T=0" supplemental images to the "T=2 h" shown in Figure 4 to capture the start of internalization. Figure shows that before internalization, all tested conditions (a-e) had similar AF647 signals, demonstrating that when one of the epitopes was blocked (d-e), the anti-HER2-Bs-AF647 was bound to the cells via a second epitope without a decrease in binding (compare c, d, e). The decrease in anti-HER2-Bs-AF647 internalization in the presence of excess either trastuzumab (D) or 39S (E) was due to the blockade of the epitope, but not reduced binding of anti-HER2-Bs in the presence of a blocking antibody.

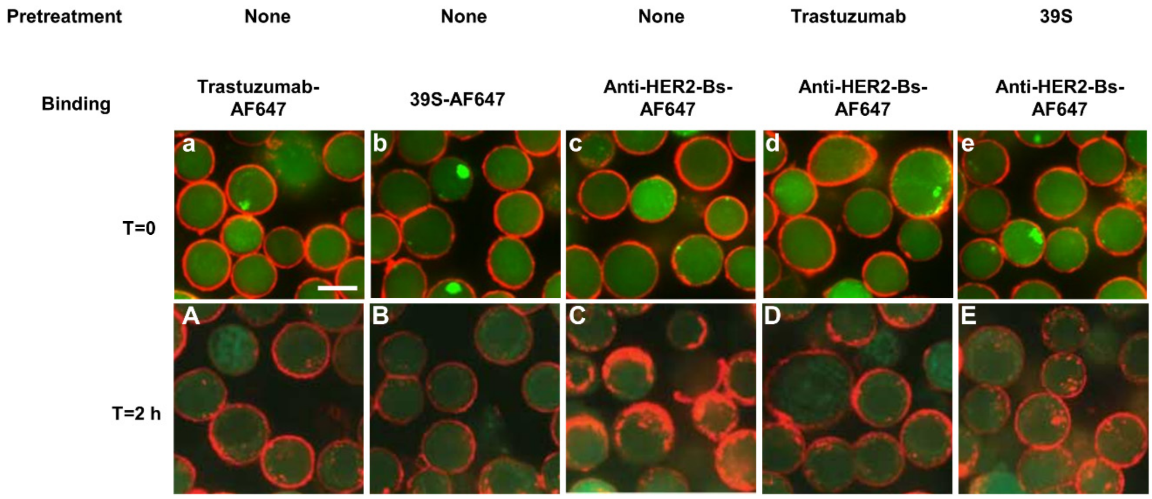

Figure S3. Blocking one epitope on HER2 receptors slowed down internalization of anti-HER2-Bs-AF647 but did not reduce its cell binding. At the start of internalization (T=0, (a-e)) all AF647-labeled antibodies show similar binding to the cells surface, indicating that blocking pre-treatment allowed full binding of the anti-HER2-Bs to surface receptors via another epitope*. Similar to the previous figures shown, anti-HER2-Bs-AF647 internalized rapidly (C) compared to either trastuzumab-AF647 (A) or 39S-AF647 (B) at 2 h. (D) By contrast, blocking of trastuzumab-binding epitopes significantly slowed down the internalization of anti-HER2-Bs-AF647 at 2 h (compare D to C) and resembled the internalization of 39S alone (compare D to B). (E) Similarly, blocking of the other epitopes (39S-binding epitopes) also slowed down the anti-HER2-Bs-AF647 internalization (compare E to C). Scale bar is 10 μm.

*Note, that 100X excess of each unlabeled competitor was used in blocking experiments. These concentrations were confirmed to fully block AF647-labeled antibodies (tratuzumab-AF647, 39S-AF647 or anti-HER2-Bs-AF647) used in binding experiments.

Supplementary materials to Figure 6 (Section 3.5.1 "Internalized anti-HER2-Bs stayed associated with HER2 while internalized trastuzumab and 39S dissociated from HER2")

Trafficking pathways of internalized HER2 receptors mediated by anti-HER2-Bs and parental antibodies are shown in Figure S4. Trastuzumab and 39S exerted similar effects on HER2 trafficking, whereas anti-HER2-Bs mediated a different intracellular pathway.

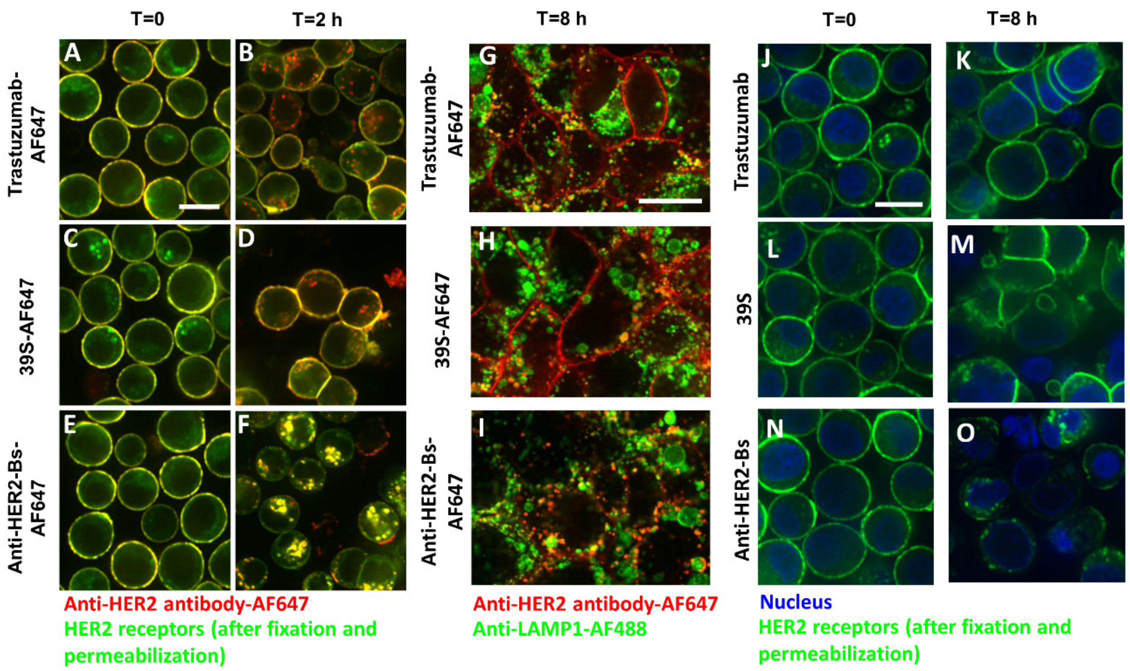

Figure S4. Anti-HER2-Bs and parental arm antibodies (trastuzumab and 39S) mediated distinct intracellular trafficking of internalized HER2 receptors. Internalization of trastuzumab-AF647 (A-B), 39S-AF647 (C-D) anti-HER2-Bs-AF647 (E-F) are shown. Before the initiation of internalization (A, C, E), both trastuzumab-AF647 (red), 39S-AF647 (red) and anti-HER2-Bs-AF647 (red) were colocalized with HER2 receptors on the cell surface (green) and appeared yellow. Upon internalization at 2 h anti-HER2-Bs-AF647 and parental arm antibodies: trastuzumab-AF647 and 39S-AF647 mediated strikingly different trafficking of HER2 receptors. Internalized anti-HER2-Bs-AF647 (red) remained colocalized with HER2 receptors (green) and appeared as yellow puncta (F). However, trastuzumab-AF647 (B) and 39S-AF647 (D) were dissociated from HER2 receptors and appeared as red puncta in the cells. Treatment with anti-HER2-Bs (N, O) resulted in complete surface clearance of HER2 receptors at 8 h, whereas neither trastuzumab (J, K), nor 39S (L, M) cleared surface HER2. Internalized anti-HER2-Bs-AF647 (I), trastuzumab-AF647 (G) and 39S-AF647 (H) are colocalized with lysosomal marker, LAMP1-AF488, at 8 h. The red puncta of internalized anti-HER2-Bs-AF647 (I) or trastuzumab-AF647 (G) or 39S-AF647 (H), colocalized with the LAMP1-AF488-positive compartments (green) merging as yellow. Treatment of anti-HER2-Bs resulted in the complete clearance of the antibody from the cell surface by 8 h (O). By contrast, there was a significant amount of trastuzumab-AF647 (K) or 39S-AF647 (M) remaining on the cell surface after treatments. Scale bars are 10 µm.
